# Impostor Phenomenon Measurement Scales: A Systematic Review

**DOI:** 10.3389/fpsyg.2019.00671

**Published:** 2019-04-05

**Authors:** Karina K. L. Mak, Sabina Kleitman, Maree J. Abbott

**Affiliations:** School of Psychology, University of Sydney, Sydney, NSW, Australia

**Keywords:** impostor phenomenon, impostorism, validation, measure, psychometric

## Abstract

The impostor phenomenon is a pervasive psychological experience of perceived intellectual and professional fraudulence. It is not a diagnosable condition yet observed in clinical and normal populations. Increasingly, impostorism research has expanded beyond clinical and into applied settings. However, to date, a systematic review examining the methodological quality of impostorism measures used to conduct such research has yet to be carried out. This systematic review examines trait impostor phenomenon measures and evaluates their psychometric properties against a quality assessment framework. Systematic searches were carried out on six electronic databases, seeking original empirical studies examining the conceptualization, development, or validation of self-report impostor phenomenon scales. A subsequent review of reference lists also included two full-text dissertations. Predetermined inclusion and exclusion criteria were specified to select the final 18 studies in the review sample. Of the studies included, four measures of the impostor phenomenon were identified and their psychometric properties assessed against the quality appraisal tool—Clance Impostor Phenomenon Scale, Harvey Impostor Scale, Perceived Fraudulence Scale, and Leary Impostor Scale. The findings often highlighted that studies did not necessarily report poor psychometric properties; rather an absence of data and stringent assessment criteria resulted in lower methodological ratings. Recommendations for future research are made to address the conceptual clarification of the construct's dimensionality, to improve future study quality and to enable better discrimination between measures.

## Introduction

The impostor phenomenon describes a psychological experience of intellectual and professional fraudulence (Clance and Imes, 1978; Matthews and Clance, 1985). Individuals experiencing impostorism believe others have inflated perceptions of their abilities and fear being evaluated. Thus, they fear exposure as “frauds” with a perceived inability to replicate their success. This fear exists despite evidence of on-going success. Such individuals also discount praise, are highly self-critical and attribute their achievements to external factors such as luck, hard work or interpersonal assets, rather than internal qualities such as ability, intelligence or skills (Harvey, 1981; Matthews and Clance, 1985).

The phrase impostor phenomenon first appeared in the late 1970's following clinical observations of female clients (Clance and Imes, 1978). A Google Scholar search returns over 1,200 impostor phenomenon scholarly publications since 1978. Over 80% of these papers are publications from impostorism research conducted in the last 20 years. Mainstream publications (e.g., Harvard Business Review) have also dedicated articles spotlighting the impostor phenomenon and how to “deal with” or “overcome” this psychological experience (e.g., Molinsky, 2016; Stahl, 2017; Wong, 2018). TED talks viewed over 14 million times online offer body language solutions and the notion of faking it until you make it to overcome the impostor “syndrome” (Cuddy, 2012). While mainstream media, has offered solutions to this psychological experience, peer-reviewed literature identify variations in definitions and conceptualizations of trait impostorism (Sakulku, 2011). Increasingly, systematic literature reviews are commonly being carried out to evaluate validation studies of self-report measurement scales, for example, anxiety or resilience measures (Windle et al., 2011; Modini et al., 2015). However, to date, a systematic literature review examining the methodological quality of impostor phenomenon measures has yet to be conducted. This is a significant gap given the increased research and mainstream interest in the impostor phenomenon. Researchers and practitioners rely on psychometrically robust measures to draw meaningful interpretations of data and to offer individuals the most appropriate evidenced-based solutions to successfully manage this experience.

### Definitions of the Impostor Phenomenon

The impostor phenomenon was originally observed in clinical female populations and defined as a predisposition unique to successful individuals (Clance and Imes, 1978). However, Harvey (1981) asserted a failure to internalize success and viewing oneself as an impostor was not limited to highly successful people. Rather, impostorism is experienced when individuals are specifically faced with achievement tasks regardless of their success status or gender (Harvey and Katz, 1985). Furthermore, anticipation and exposure to achievement tasks are associated with negative emotions and self-beliefs such as anxiety, depression and low self-esteem among individuals experiencing impostorism (Cozzarelli and Major, 1990; Chrisman et al., 1995).

One conceptualization of the impostor phenomenon is referred to as perceived fraudulence (Kolligian and Sternberg, 1991). Similar to previous descriptions, this construct is conceptualized as multidimensional and characterized by fraudulent ideation, self-criticism, achievement pressure and negative emotions. However, perceived fraudulence also emphasizes impression management and self-monitoring by individuals who are concerned about their self-worth and social image; constructs not emphasized in previous definitions. Kolligian and Sternberg (1991) also emphasize that rather than being a unitary personality disorder, the imposter phenomenon is better represented by the term “perceived fraudulence,” since it alludes to a self-critical outlook, the illusion of fraudulence and a strong focus on vigilant impression management (Kolligian and Sternberg, 1991).

Leary et al. (2000) acknowledge the three key attributes of traditional definitions of the impostor phenomenon—the sense of being a fraud, fear of being discovered and difficulty internalizing success while behaving in ways that maintain these beliefs. However, they argue these central characteristics are paradoxical, especially the belief impostors hold of others overestimating their intelligence or ability. Studies have shown discrepancies between self- and reflected appraisals in individuals experiencing impostorism and found differences in how impostors react when their responses are public vs. private and when the other person (“perceiver”) is seen as equal or higher in status (Leary et al., 2000; McElwee and Yurak, 2007, 2010). This alludes to a self-presentation characteristic similar to Kolligian and Sternberg (1991), however, Leary et al. (2000) instead focus on the core feeling of inauthenticity as being central to the conceptualization of impostorism. Unlike previous definitions and measures, a unidimensional definition is adopted and solely focuses on feeling like a fraud among many individuals, not just successful people.

### Aims

The primary aims of the present review are to (1) systematically identify self-report measures of the impostor phenomenon in the literature, (2) assess the psychometric properties presented in validation studies against a standardized quality appraisal tool, (3) discuss the conceptualization of the construct against an evaluation of the usefulness of the identified measures and (4) ascertain whether a gold standard measure of the impostor phenomenon exists. The review will also follow the PRISMA Statement and guidelines for conducting and reporting systematic reviews (Liberati et al., 2009).

### Measures of the Impostor Phenomenon

Different definitions of the impostor phenomenon have led to the development of various measurement scales for clinical and research applications. The first instrument was constructed by Harvey (1981), a 14-item scale developed with graduate and undergraduate populations. Subsequently, the Clance Impostor Phenomenon Scale was developed (Clance, 1985) to improve measurement of the impostor phenomenon and to better account for clinically observed attributes or feelings not addressed by the Harvey Impostor Scale. Unlike the Harvey Impostor Scale, this 20-item instrument acknowledges the fear of evaluation and feeling less capable than peers. It is also positively worded to minimize social desirability effects. The Clance Impostor Phenomenon Scale is the most commonly used measure by researchers and practitioners. Despite this popularity, research is yet to firmly establish the strength of this instrument over others.

Other measures such as the Perceived Fraudulence Scale (Kolligian and Sternberg, 1991) and Leary Impostor Scale (2000) also reflect the researchers' respective definitions of the construct. The 51-item Perceived Fraudulence Scale reflects the multidimensional and impression managing characteristics outlined by Kolligian and Sternberg (1991). In comparison, the Leary Impostor Scale is a 7-item instrument aligned to a unidimensional conceptualization of the impostor phenomenon as solely focused on a sense of being an impostor or fraud (Leary et al., 2000). Despite the variation in definition and popularity of some measures over others, these instruments are yet to be subjected to a systematic evaluation of their psychometric properties.

This review will focus on evaluating the quality of impostor phenomenon measures against criteria from a published measurement quality framework (Terwee et al., 2007). It will leverage definitions from the Standards for Educational and Psychological Testing (American Educational Research Association et al., 2014) to ensure consistency with current psychometric guidelines for scale validation. The current validation studies of impostor phenomenon measures have focused on Clance (1985) and Harvey's (1981) scales, with minimal evaluation of the Perceived Fraudulence Scale (Kolligian and Sternberg, 1991) and Leary Impostor Scale (Leary et al., 2000). This review aims to address this gap. Theoretically, each measure reflects the features of each definition. Harvey (1981), Clance (1985), and Kolligian and Sternberg (1991) postulate that impostorism is a multidimensional construct. However, the authors have outlined different dimensions. In contrast, Leary et al. (2000) focus on a unidimensional definition. Collectively, these measures will be the focus of this systematic review.

### Relationships to Other Variables

From these instruments, the impostor phenomenon has been examined in relation to demographic variables, personality and recently, workplace outcomes. Impostorism affects both genders (e.g., Harvey, 1981; Topping and Kimmel, 1985), different ethnic backgrounds (Chae et al., 1995), and occupations (e.g., Want and Kleitman, 2006; Bechtoldt, 2015). The construct is also associated with maladaptive perfectionism (Ferrari and Thompson, 2006) engagement in self-handicapping behaviors (Want and Kleitman, 2006) and lowered well-being outcomes (Chrisman et al., 1995).

Recent studies in the workplace have highlighted the impact of impostorism on relevant work attitudes and behaviors. Stronger impostorism feelings in working professionals are associated with lower levels of job satisfaction, lower organizational citizenship behaviors—discretionary actions that benefit colleagues and the organization—and higher continuance commitment, that is, higher perceived costs of leaving their organization (Vergauwe et al., 2015). These findings suggest the impostor phenomenon has consequences beyond clinical and student populations. In addition, integral to theory development is the ability to differentiate a construct from its antecedents and outcomes. Therefore, developing a thorough understanding of the nature of the impostor phenomenon and its consequences requires the use of psychometrically sound and appropriate tools to measure the construct.

To date, a published study systematically reviewing research on the psychometric properties of impostor phenomenon measures has not been conducted. This is a significant gap given the increased research interest beyond clinical and academic settings. The validity of research findings is conditional on the use of the most valid, reliable and appropriate tools measuring constructs of interest. Therefore, identifying psychometrically robust instruments through a systematic review is justified. This will be an important contribution to the current evidence base and support the meaningful interpretation of results that have real-world implications.

## Methods

### Search Strategy

A systematic search was conducted in six electronic databases—PsycINFO, Web of Science, Business Source, Scopus, Proquest and Cochrane Database of Systematic Reviews. Peer-reviewed journal articles, book chapters, and subsequently dissertations that focused on defining, conceptualizing and validating self-report impostor phenomenon measures through empirical studies in the English language were sought. Reference lists of all included studies were also manually screened for potentially relevant publications.

Relevant studies were identified using a combination of keywords and phrases relating to the impostor phenomenon (e.g., “impostor phenomenon,” “impostorism,” “impostor syndrome,” a variation in spelling of “imposter” and “perceived fraudulence”), self-report measures (e.g., “questionnaire,” “measurement,” “assessment”), and validation (“validate,” “validation,” “psychometric”). The final search was conducted in all databases on 22nd February 2018. First authors were contacted for further information regarding papers not accessible through databases with limited success.

#### Inclusion Criteria

Peer-reviewed journal articles and unpublished dissertations were included in the review if they were an original quantitative research study that developed, validated and/or investigated the psychometric properties of a self-report measure of trait impostorism and sampled an adolescent or adult population. Only studies published in the English language were included which also included studies conducted on non-English speaking samples, as long as the research was based on trait impostor phenomenon measures.

#### Exclusion Criteria

Studies were excluded in the review if a child population was utilized, were non-peer reviewed journal articles, conference proceedings, non-psychometric studies and not written in the English language. It was noted, there are currently no evidence-based interventions for the impostor phenomonenon and as a result, comparators or outcomes in the literature to be accounted for by this systematic review. Therefore, this review has been limited to comprehensively defining the populations of interest and specific study designs in the inclusion and exclusion criteria.

### Selection Process

Search results were initially screened by title and abstract to exclude research that did not meet the inclusion criteria. Subsequently, of the remaining studies, the full-text papers were obtained and evaluated according to their relevance in meeting the stipulated inclusion/exclusion criteria.

### Data Extraction and Quality Assessment

The evaluation of scales was guided by definitions and principles presented in the Standards for Educational and Psychological Testing (American Educational Research Association et al., 2014). Specifically, validity was viewed as a unitary concept and the extent to which different types of accumulated validity evidence supported the intended interpretation of test scores. Reliability was concerned with reliability coefficients of classical test theory and the consistency of scores across replications (American Educational Research Association et al., 2014). The psychometric properties of all included studies were assessed by applying a published quality appraisal tool (Terwee et al., 2007). This comprehensive quality assessment framework considers the domains of validity, reliability, and responsiveness. It is typically applied to evaluate the measurement quality of health-status questionnaires. Although the impostor phenomenon is not an officially diagnosable health condition, its measures are similar to health-status instruments and designed to identify individuals who self-report experiencing the phenomenon, which in itself, is associated with established well-being consequences and poorer mental health (e.g., Chrisman et al., 1995; Sonnak and Towell, 2001). Based on these conditions, this measurement framework was considered appropriate to evaluate studies examining the psychometric properties of impostor phenomenon measures (Terwee et al., 2007). The nine measurement properties appraise content validity, internal consistency, construct validity, reproducibility: agreement, reproducibility: reliability, responsiveness, floor or ceiling effects and interpretability. The definitions and criteria of adequacy for each psychometric property are displayed in Table 1. Specific criteria from the original framework were only applied to certain papers due to the limited number of validation studies and noted in Table 1. For example, the assessment framework (Terwee et al., 2007) classifies item selection as relevant criteria for content validity, however, this review only considered item selection as a compulsory and applicable criterion for original scale development studies.

**Table 1:** Criteria for adequacy of psychometric properties and scoring system (Terwee et al., 2007).

	**Property**	**Definition**	**Quality criteria**		
1	Content validity	The extent to which the domain of interest is comprehensively sampled by the items in the questionnaire (the extent to which the measure represents all facets of the construct under question).	+	2	A clear description of measurement aim, target population, concept(s) that are being measured, and the item selection^*^ AND target population and (investigators OR experts) were involved in item selection
			?	1	A clear description of above-mentioned aspects is lacking OR only target population involved OR doubtful design or method
			–	0	No target population involvement
			0	0	No information found on target population involvement
2	Internal consistency	The extent to which items in a (sub)scale are inter-correlated, thus measuring the same construct	+	2	Factor analyses performed on adequate sample size (7^*^ #items and > = 100) AND Cronbach's alpha(s) calculated per dimension^**^ AND Cronbach's alpha(s) between 0.70 and 0.95
			?	1	No factor analysis OR doubtful design or method
			–	0	Cronbach's alpha(s) <0.70 or >0.95, despite adequate design and method
			0	0	No information found on internal consistency
3	Criterion validity	The extent to which scores on a particular questionnaire relate to a gold standard	+	2	Convincing arguments that gold standard is “gold” AND correlation with gold standard > = 0.70
			?	1	No convincing arguments that gold standard is “gold” OR doubtful design or method
			–	0	Correlation with gold standard <0.70, despite adequate design and method
			0	0	No information found on criterion validity
4	Construct validity	The extent to which scores on a particular questionnaire relate to other measures in a manner that is consistent with theoretically derived hypotheses concerning the concepts that are being measured	+	2	Specific hypotheses were formulated AND at least 75% of the results are in accordance with these hypotheses
			?	1	Doubtful design or method (e.g., no hypotheses)
			–	0	< 75% of hypotheses were confirmed, despite adequate design and methods
			0	0	No information found on construct validity
5	Reproducibility: Agreement	The extent to which the scores on repeated measures are close to each other (absolute measurement error)	+	2	SDC < MIC OR MIC outside the LOA OR convincing arguments that agreement is acceptable
			?	1	Doubtful design or method OR (MIC not defined AND no convincing arguments that agreement is acceptable)
			–	0	MIC < = SDC OR MIC equals or inside LOA despite adequate design and method
			0	0	No information found on agreement
6	Reproducibility: Reliability	The extent to which patients can be distinguished from each other, despite measurement errors (relative measurement error)	+	2	ICC or weighted Kappa > = 0.70
			?	1	Doubtful design or method
			–	0	ICC or weighted Kappa <0.70, despite adequate design and method
			0	0	No information found on reliability
7	Responsiveness	The ability of a questionnaire to detect clinically important changes over time	+	2	SDC or SDC < MIC OR MIC outside the LOA OR RR > 1.96 OR AUC > = 0.70
			?	1	Doubtful design or method
			–	0	SDC or SDC > = MIC OR MIC equals or inside LOA OR RR < = 1.96 or AUC <0.70, despite adequate design and methods
			0	0	No information found on responsiveness
8	Floor and ceiling effects	The number of respondents who achieved the lowest or highest possible score	+	2	=<15% of the respondents achieved the highest or lowest possible scores
			?	1	Doubtful design or method
			–	0	>15% of the respondents achieved the highest or lowest possible scores, despite adequate design and methods
			0	0	No information found on interpretation
9	Interpretability	The degree to which one can assign qualitative meaning to quantitative scores	+	2	Mean and SD scores presented of at least four relevant subgroups of patients and MIC defined
			?	1	Doubtful design or method OR less than four subgroups OR no MIC defined
			–	0	No information found on interpretation

In order to calculate a total score + = 2 positive rating; ? = 1 indeterminate rating; – = 0 negative rating; 0 = no information available.

Total score range 0–18.

RR, responsiveness ratio.

* Item selection criterion only applied to original scale development studies.

** Cronbach's alpha calculated per dimension if the impostor phenomenon is conceptualized as multidimensional in the specific study.

SDC, Smallest detectable difference (this is the smallest within person change, above measurement error. A positive rating is given when the SDC or the limits of agreement are smaller than the MIC).

MIC, Minimal important change (this is the smallest difference in score in the domain of interest which patients perceive as beneficial and would agree to, in the absence of side effects and excessive cost)s.

SEM, standard error of measurement;

*AUC, area under the curve*.

Each category received evaluative ratings and scores of “+” as good, “?” for being intermediately rated, “–” for being negatively rated or a “0” was assigned if no information was provided on that criterion in a specific study. A “Not Reported” (NR) rating was also allocated for properties not exclusively addressed in the studies. Unlike Terwee et al.'s (2007) framework, this review also provides an overall methodological total score for each study. This total score is not a marker of overall quality, however, it provides a metric to rank the 18 studies selected in this review and to aid researchers and practitioners with their unique objectives. The ratings on each measurement property were totaled across all studies from low (0) to high (18).

Two researchers independently evaluated each included study and rated their psychometric and methodological quality against the quality framework. Discrepancies in scoring were discussed at calibration meetings to arrive at a consensus.

## Results

**Figure 1:**
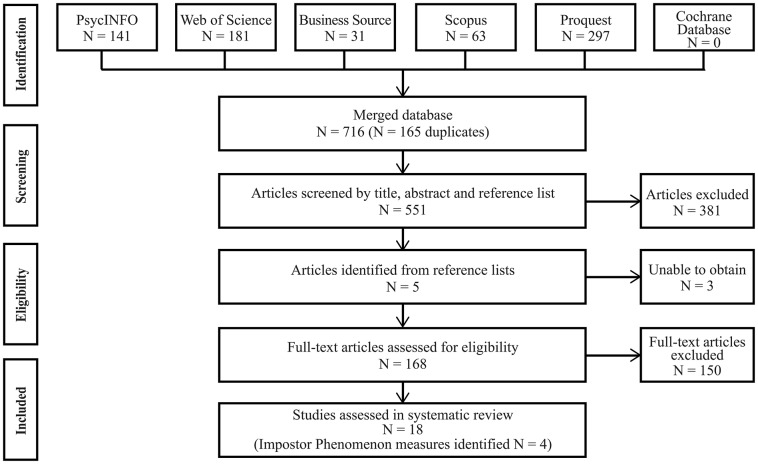
Flow diagram of study selection.

The initial search returned 716 potential studies, of which 165 were duplicates. Studies were most commonly excluded for not being a validation study, not reporting psychometric data on an impostor phenomenon measure, using a child sample or not published in the English language. Figure 1 is a flow diagram documenting the review process. Overall, 18 studies were evaluated in this systematic review. Initially, 16 articles met the inclusion criteria. Subsequently, an additional two studies were included following review of reference lists. Despite these two studies being unpublished doctoral dissertations, a decision was made to include this research due to the limited number of validation studies available. One dissertation was the original scale development study for the Harvey Impostor Scale (Harvey, 1981) and the second, an often cited validation study in peer-reviewed articles (Topping, 1983). The authors noted Topping and Kimmel (1985) published an abbreviated version of results from Topping's (1983) unpublished dissertation. The authors of this review decided to only evaluate the Topping (1983) dissertation as it included the full set of results from the sample of 285 university faculty members. Overall 4 impostor phenomenon measures were identified—Clance Impostor Phenomenon Scale (CIPS; Clance, 1985), Harvey Impostor Scale (HIPS; Harvey, 1981), Perceived Fraudulence Scale (PFS; Kolligian and Sternberg, 1991), and Leary Impostorism Scale (LIS; Leary et al., 2000). Of the 18 studies included in the systematic review, 11 primarily examined the CIPS, 5 examined the HIPS, 1 examined the PFS, and 1 examined the LIS. Table 2 describes the studies included in this review organized by measure and ascending year of publication.

**Table 2:** Included study descriptions.

**Questionnaire**	**Original study (Y/N)**	**Factor analysis or IRT**	**Number of items**	**Study population #**	**Age (Mean)**	**Sex ratio**
**CLANCE IMPOSTOR PHENOMENON SCALE**						
Cozzarelli and Major, 1990	N	Correlations, ANOVA and MANOVA	20 CIPS	137 undergraduates	Not reported	85 females and 52 males
Holmes et al., 1993	Y	Correlations, ANOVA, ANCOVA and regression modeling	20 CIPS and 14 HIPS	62 subjects (32 clinical and 30 nonclinical)	28.9 years *SD* = 8.6	48 females and 14 males
Chae et al., 1995	Y	Correlations and *t-*tests	20 Korean CIPS	640 Korean Catholics	34 years *SD* = 10.7	334 females and 320 males
Chrisman et al., 1995	Y	Factor analysis with PCA. 3 factor model—fake, discount and luck when items 1, 2, 19, and 20 were excluded	20 CIPS and 51 PFS	269 undergraduates	23 years *SD* not reported	69% female ~31% male
French et al., 2008	N	CFA with RWLS estimation. Model 1–3 factors (theoretically preferred i.e., fake, luck and discount) and Model 2 - 2 factors (fake and discount collapsed) - best fit.	16 CIPS (4 items removed from original CIPS based on low discrimination from unpublished manuscript Kertay et al., 1992)	1,271 Engineering undergraduates	18.22 years *SD* = 0.86	242 females and 1,029 males
McElwee and Yurak, 2010	Y	PCA. Chrisman et al. (1995) 3 factors Fear, Luck and Discount. Then ANOVA and correlations to examine relationships between and across groups	20 CIPS and 7 LIS	122 undergraduates in psychology courses	19.64 years *SD* = 3.01	90 female and 32 male
Jöstl et al., 2012	Y	CFA of German CIPS and then path modeling for several related regression relationships—replicated Chrisman et al.'s (1995) 3-factor model with poor fit. Single factor model of CIP better fit.	16 German CIPS (items modified for doctoral students)	631 Austrian doctoral students	31.5 years *SD* = 7.8	389 females and 242 males
Rohrmann et al., 2016	N	CFA	20 CIPS	242 people occupying leadership positions from Germany	44.3 years *SD* = 9.02	37% female
Brauer and Wolf, 2016	Y	EFA with Sample 1 and CFA with Sample 2 resulting in 3 factor model - fake, discount and luck	20 German CIPS	Study 1: 151 mostly undergraduates and school leaving diploma graduates Study 2: 149 psychology undergraduates	Study 1: 27.5 years *SD* = 10.3 Study 2: 22.5 years *SD* = 4.5	Sample 1: 113 females and 38 males Sample 2: 113 females and 36 males
Leonhardt et al., 2017	Y	Agglomerative cluster analysis with the Ward procedure to distinguish between groups who experience impostorism. 2 clusters and then compared with *t*-tests	20 German CIPS	183 employees in leading positions	44.3 years *SD* = 9.02	36.77% female
Simon and Choi, 2018	N	CFA with best fitting one factor model	20 CIPS	211 Doctoral students from STEM fields	72% of respondents in the 20–30 age category. *SD* not reported	108 females and 103 males
**HARVEY IMPOSTOR PHENOMENON SCALE**						
Harvey, 1981^*^	Y	Correlations and independent samples *t-*test. ANOVA	14 HIPS	Study 1: 74 graduate students Study 2: 72 undergraduates (for cross-validation)	Not reported	Not reported
Topping, 1983^*^	N	Correlations, *t*-tests, ANOVA	14 HIPS	285 university faculty members	Not reported	157 females and 128 males
Edwards et al., 1987	N	Factor analysis with principal axis factoring. Proposes a three factor model - impostor, unworthiness, inadequacy	14 HIPS	104 postgraduates receiving training or already received advanced degrees	males (*M* = 31.9 years, *SD* = 4.4) females (*M* = 34.8 years, *SD* = 9.5)	78 females and 26 males
Fried-Buchalter, 1992	N	Correlations and factor analysis using PCA followed by oblique and orthogonal varimax rotations. First and second-order factor analyses.	14 HIPS	104 mid-level marketing managers from New York	Not reported	51 females and 53 males
Hellman and Caselman, 2004	N	Factor analysis with PCA. Proposes a 2 factor model from 9 items – self-confidence and impostorism	14 HIPS	136 high school adolescents	Not reported	52.2% female 48.4% male
**PERCEIVED FRAUDULENCE SCALE**						
Kolligian and Sternberg, 1991	Y	Factor analysis with PCA and varimax rotation on both studies. Proposes 2 factor model of perceived fraudulence - self-deprecation and inauthenticity Stepwise regression analysis.	51 PFS (and 14 HIPS in Study 1)	Trial Study: 60 Yale undergraduates to develop PFS (results available on request; not focus of the study) Study 1: 50 undergraduates Study 2: 100 undergraduates	Study 1: *M* = 18.36 years, *SD* = 0.96 Study 2: *M* = 18.46 years, *SD* = 1.23	Study 1: 26 males and 24 females Study 2: 50 males and 50 females
**LEARY IMPOSTOR SCALE**						
Leary et al., 2000	Y	Type of analysis not reported in scale development of LIS 7 item unidimensional measure “capturing the essence of impostorism i.e. of being an impostor or fraud.”	7 LIS (in studies 1–3) 14 HIPS and 20 CIPS (Study 2)	Study 1: 238 undergraduates Study 2: 95 undergraduates Study 3: 67 undergraduates	Only range 17–23 years reported	Study 1:119 females and 119 males Study 2: 47 females and 48 males Study 3: not reported (*N* = 67)

CFA, confirmatory factor analysis; EFA, exploratory factor analysis; IRT, item response theory; PCA, principal component analysis; RWLS, robust weighted least squares estimation; CIPS, Clance Impostor Phenomenon Scale; HIPS, Harvey Impostor Scale; PFS, Perceived Fraudulence Scale; LIS, Leary Impostor Scale; SD, Standard Deviation.

* *Unpublished dissertation*.

### Assessment of Psychometric Properties

The assessment of psychometric properties was conducted using measurement criteria defined by Terwee et al. (2007) and leveraged the principles from the Standards for Educational and Psychological Testing (American Educational Research Association et al., 2014). Two observers independently rated each included study against the nine psychometric properties of the quality appraisal tool (Terwee et al., 2007). Agreement between the two reviewers on criteria of adequacy was 80% and this equates to a Kappa of *k* = 0.66 (*p* <0.000). Kappa is an inter-rater agreement statistic that controls for the agreement expected based on chance alone and a kappa of 0.66 represents a substantial degree of agreement between raters (Cohen, 1960).

The impostor phenomenon measures in each study were assessed against the nine measurement categories from Terwee et al.'s (2007) appraisal tool outlined in Table 1. The following evaluative ratings and scores were applied—“+” (2) as good, “?” (1) as intermediately rated, “–” (0) negatively rated or a “0” (0) was assigned if no information was available. A “Not Reported” (NR) rating was also allocated for properties not exclusively addressed in the studies. A fifth rating was also introduced and applied exclusively to Criterion Validity—“Currently Not Possible” (CNP). This rating reflected the limited evidence base in which a “gold standard” comparison was not possible and therefore applied to Criterion Validity. Although an overall score is not stipulated in the assessment framework, the ratings on each measurement property were totaled across all studies from low (0) to high (18) (refer to Table 3). It is important to note this was not an overall quality score, rather a useful metric to rank the reviewed studies.

**Table 3:** Overview of ratings on psychometric properties in included studies.

**Questionnaire**	**Content validity**	**Internal consistency**	**Criterion validity**	**Construct validity**	**Reproducibility: Agreement**	**Reproducibility: Reliability**	**Responsiveness**	**Floor and ceiling effects**	**Interpretability**	**Total**
**CLANCE IMPOSTOR PHENOMENON SCALE**										
Cozzarelli and Major, 1990	+	?	CNP	?	NR	NR	NR	0	?	
	2	1		1				0	1	5
Holmes et al., 1993	+	?	CNP	?	NR	NR	NR	+	+	
	2	1		1				2	2	8
Chae et al., 1995	+	?	CNP	?	NR	NR	NR	0	0	
	2	1		1				0	0	4
Chrisman et al., 1995	?	?	CNP	?	NR	NR	NR	0	?	
	1	1		1				0	1	4
French et al., 2008	+	+	CNP	?	NR	NR	NR	0	?	
	2	2		1				0	1	6
McElwee and Yurak, 2010	+	?	CNP	?	NR	NR	NR	0	0	
	2	1		1				0	0	4
Jöstl et al., 2012	+	+	CNP	0	NR	NR	NR	0	+	
	2	2		0				0	2	6
Rohrmann et al., 2016	+	?	CNP	+	NR	NR	NR	0	?	
	2	1		2				0	1	6
Brauer and Wolf, 2016	?	?	CNP	+	NR	NR	NR	+	?	
	1	1		2				2	1	7
Leonhardt et al., 2017	+	?	CNP	+	NR	NR	NR	0	?	
	2	1		2				0	1	6
Simon and Choi, 2018	?	+	CNP	?	NR	NR	NR	0	0	
	1	2		1				0	0	4
**HARVEY IMPOSTOR PHENOMENON SCALE**										
Harvey, 1981^*^	+	?	CNP	+	NR	NR	NR	0	?	
	2	1		2				0	1	6
Topping, 1983^*^	+	?	CNP	0	NR	NR	NR	+	+	
	2	1		0				2	2	7
Edwards et al., 1987	?	?	CNP	0	NR	NR	NR	+	?	
	1	1		0				2	1	5
Fried-Buchalter, 1992	+	?	CNP	+	NR	NR	NR	0	?	
	2	1		2				0	1	6
Hellman and Caselman, 2004	+	?	CNP	?	NR	NR	NR	0	?	
	2	1		1				0	1	5
**PERCEIVED FRAUDULENCE SCALE**										
Kolligian and Sternberg, 1991	+	?	CNP	?	NR	NR	NR	0	?	
	2	1		1				0	1	5
**LEARY IMPOSTOR SCALE**										
Leary et al., 2000	?	?	CNP	+	NR	NR	NR	0	?	
	1	1		2				0	1	5

* Unpublished dissertation.

*“+” (2) = good, “?” (1) = intermediate, “-” (0) = negative, “0” (0) = no information available. NR, “Not Reported” not exclusively addressed in the study; CNP, “Currently Not Possible” insufficient evidence base to establish “gold standard” comparison*.

#### Content Validity

All studies provided adequate evidence of the measurement aim, target population, and concepts being measured. The assessment framework (Terwee et al., 2007) classifies item selection as relevant criteria for content validity, however, this review only considered item selection as a compulsory criterion for original scale development studies of which there were only three studies. Harvey's (1981) study was rated positively for content validity because item selection was driven by theoretical and therapeutic observations, and reported item analysis statistics. Kolligian and Sternberg's (1991) study also received a positive rating for sufficient item selection information however, Leary et al.'s (2000) article was rated indeterminate overall for content validity as Study 1 provided adequate evidence, however, Study 2 did not provide item selection information for the LIS development. The PFS and LIS were newly developed impostor phenomenon measures at the time and scale development data was only available upon request from the first authors. Responses from first authors had not been received by the time of submission for this publication. Two other studies were also allocated an indeterminate rating for content validity. Simon and Choi (2018) and Brauer and Wolf's (2016) studies provided brief measurement aims, explanations for the constructs of interest and little to no justification for the target populations sampled.

#### Internal Consistency

Three studies received positive ratings for internal consistency. These studies conducted factor analysis on an adequate sample size, with appropriate design and method, and reported Cronbach's alphas between 0.70 and 0.95 for each dimension and overall. The remaining studies were allocated indeterminate ratings for gaps in addressing one or more criterion for internal consistency. Item Response Theory analysis was also an acceptable form of analysis for the internal consistency criterion, however, none of the selected studies in the review sample carried out this form of analysis.

Sixteen studies reported Cronbach alpha scores of adequate magnitude for the impostor phenomenon measures. Among the 11 CIPS studies, overall Cronbach alphas ranged from 0.85 to 0.96. Seven of these studies examined the factorial structure of the CIPS and only three reported the subscale reliability statistics (French et al., 2008; McElwee and Yurak, 2010; Brauer and Wolf, 2016). Cronbach alphas were presented for factors in a theoretically preferred three factor model for the CIPS—Fake (0.84), Discount (0.79), and Luck (0.70), compared to a statistically better fitting two factor model, however, subscale reliabilities were not reported for the two factor model (French et al., 2008). Similarly, a three factor model was replicated for the CIPS with subscale reliabilities ranging from 0.74 to 0.89 (McElwee and Yurak, 2010). A third study validated the German CIPS (0.87–0.89) utilizing exploratory and confirmatory factor analysis with two samples (Brauer and Wolf, 2016). A three factor model resulted in the best fit statistics and Cronbach alphas for each factor Fake (0.84), Discount (0.73), and Luck (0.69). This three factor structure aligned to the typical three characteristics of the impostor phenomenon presented by Clance (1985) and equivalent to the English version of the CIPS—feeling like a fake, discounting achievement, and attributing success to luck.

Five studies primarily examined the HIPS with overall Cronbach alphas ranging from 0.34 to 0.85, in addition to a study comparing the CIPS and HIPS (α = 0.91) (Holmes et al., 1993) and a second study comparing the PFS to HIPS (α = 0.64) (Kolligian and Sternberg, 1991). Three HIPS studies explored the factorial structure of the measure. One study proposed a three factor model with subscale reliabilities between 0.65 and 0.81 (Edwards et al., 1987). In comparison, a HIPS four-factor model was presented with moderate correlations however subscale alphas were not reported (Fried-Buchalter, 1992). In an adolescent sample, Hellman and Caselman (2004) reported an alpha of 0.70 for the original 14 items. However, following factor analysis, an alpha of 0.80 was reported for a better fitting 11-item two factor model (self-confidence and impostor phenomenon) for the HIPS. The subscale reliabilities were not presented.

The original PFS validation study proposed a two factor model with an overall Cronbach alpha of 0.94 and subscale reliabilities for Inauthenticity (0.95) and Self-deprecation (0.85) (Kolligian and Sternberg, 1991). Similarly, a CIPS validation study also reported an alpha of 0.94 for the PFS. However, when the Spearman-Brown equation was applied to the 51-item PFS to reduce it to the 20-item CIPS equivalent, the estimated internal reliability of the PFS was 0.57 (Chrisman et al., 1995). Leary et al.'s (2000) Study 2 reported a Cronbach's alpha of 0.87 for the unidimensional LIS.

#### Criterion Validity

A clear “gold standard” measure of the impostor phenomenon was not ascertained. Most studies did not compare the impostorism measure utilized to a “gold standard” and, if the measure was compared to an alternate impostorism measure, limited convincing rationale was provided to establish the measure as a “gold standard.” Overall, all studies in this review were allocated a “Currently Not Possible” (CNP) rating for criterion validity. Of the reviewed studies, four studies utilized two or more impostor phenomenon measures and reported correlation coefficients. Holmes et al. (1993) reported a coefficient of .89 between the CIPS and HIPS, while Chrisman et al. (1995) reported a coefficient of 0.78 between the CIPS and PFS. Kolligian and Sternberg (1991) reported a correlation of 0.83 between the PFS and HIPS. Leary et al.'s (2000) third study reported correlation coefficients between the LIS and the HIPS, CIPS, and PFS ranging from 0.70 to 0.80 and noted the LIS “showed strong evidence on construct validity” (Leary et al., 2000, p. 735).

#### Construct Validity

The construct validity criteria require studies to formulate theory-driven specific hypotheses and at least 75% of the results are in correspondence with these hypotheses to obtain a positive rating (Terwee et al., 2007). Based on these strict criteria, six studies were evaluated with a positive rating and achieved the maximum score on this property. These studies presented specific theoretically derived hypotheses that highlighted the extent to which scores on the particular impostor phenomenon measure related to other measures in a consistent manner. Among the positively rated studies examining the CIPS, HIPS, and PFS consistent yet discriminant relationships were established with other constructs. Correlations ranged from 0.34 to 0.69. Higher impostorism was associated with constructs such as low self-esteem, low confidence, high self-monitoring, higher depressive symptoms, higher anxiety, and higher negative self-evaluations than their lower impostorism counterparts (e.g., Topping, 1983; Kolligian and Sternberg, 1991; Chrisman et al., 1995; Rohrmann et al., 2016).

However, the majority of studies were intermediately rated and two studies were poorly rated because despite formulating specific hypotheses, less than 75% of the results were in accordance with the hypotheses presented (Topping, 1983) and did not consider the impostor phenomenon in relation to other measures (Edwards et al., 1987). Studies were often allocated an intermediate rating for reasons such as not formulating hypotheses rather exploratory research questions and, not considering the extent to which impostorism measures related to other measures in a manner consistent with theoretically driven hypotheses.

#### Reproducibility: Agreement

The agreement criterion is defined as the extent scores on repeated measures are close to each other (absolute measurement error) (Terwee et al., 2007). The 18 studies in the review sample did not examine repeated measures of the impostor phenomenon, therefore no information was available for agreement. Thus, all studies were assigned an “NR” rating on this measurement property as longitudinal data was not collected.

#### Reproducibility: Reliability (Test-Retest)

Reliability (test-retest) refers to the extent individuals are distinguishable from each other, despite measurement errors (Terwee et al., 2007). Among the 18 studies, changes over time in impostor phenomenon scores were not investigated and therefore allocated not reported ratings.

#### Responsiveness

Responsiveness is considered a measurement property of longitudinal validity and defined as the ability of a measure to detect clinically important changes over time (Terwee et al., 2007). Since none of the studies selected examined longitudinal changes of the impostorism measures and the construct is not classified as a clinical condition, not reported ratings were applied for all studies.

#### Floor and Ceiling Effects

Overall, four studies reported information noting equal to or less than 15% of respondents achieved the highest or lowest possible scores on the impostor phenomenon measures utilized, or provided sufficient statistics to calculate this information and therefore, received the maximum score on this criteria (Topping, 1983; Edwards et al., 1987; Holmes et al., 1993; Brauer and Wolf, 2016). Topping (1983) reported floor and ceiling results for three faculty groups, while Holmes et al. (1993) examined the CIPS and HIPS and presented the number of participants who scored the minimum and maximum across clinical and non-clinical groups. Brauer and Wolf (2016) provided skew statistics and minimum/maximum data and Edwards et al. (1987) also reported relevant statistics to ascertain whether floor and ceiling effects were present. The remaining papers did not provide information on floor and ceiling effects and subsequently allocated low ratings.

#### Interpretability

Three studies were appraised positively for providing sufficient descriptive statistics for at least four relevant subgroups. Holmes et al. (1993) provided means and standard deviations for clinician referred and non-clinical subgroups on the CIPS, while Topping (1983) comprehensively reported descriptive statistics by gender and faculty rank on the HIPS. Jöstl et al. (2012) also reported descriptive statistics by gender and faculty membership. However, the remaining studies were allocated indeterminate or poor ratings due to inadequate information to assign qualitative meaning to quantitative findings. It was also noted, several studies did not comprehensively report descriptive statistics for sample characteristics such as mean age, standard deviations and gender breakdown (refer to Table 2).

## Discussion

Measurement scales need to demonstrate adequate psychometric properties if scores are to be trusted as valid representations of constructs. Consequently, these measures can be confidently used in research and applied settings, increase conceptual understanding, and assist in the development of evidence-based support. For these reasons, a systematic review was carried out which identified self-report measures of the impostor phenomenon, assessed the psychometric properties presented against a quality appraisal tool, and discussed the conceptualization of the construct against an evaluation of the usefulness of the identified measures. The recommendations with respect to a “gold standard measure” of the impostor phenomenon are also discussed.

Eighteen studies pertaining to four trait impostor phenomenon measures were identified and evaluated utilizing a published quality appraisal tool (Terwee et al., 2007). By applying a quality appraisal framework to each study, the findings indicated strengths and several gaps across the nine measurement properties. The Standards for Educational and Psychological Testing (American Educational Research Association et al., 2014) were leveraged when considering key issues of validity and reliability in the selected studies. Based on these outcomes, a definitive conclusion about the dimensionality of the impostor phenomenon and a gold standard measure was not determined. Overall, total scores also highlighted areas for improvement in future research design and the reporting of essential psychometric data.

### Strengths and Weaknesses

#### Dimensionality

The majority of selected studies provided adequate information for content validity and internal consistency. However, gaps were evident on several criteria. Based on the study ratings for all nine measurement properties, establishing the internal dimensionality of the impostor phenomenon could not be reached due to mixed results from the selected papers. Four impostorism scales were identified—CIPS, HIPS, PFS, and LIS—which demonstrated moderate to high internal consistency, with the exception of two HIPS studies (Edwards et al., 1987; Kolligian and Sternberg, 1991).

Seven studies utilized factor analysis to develop or validate impostor phenomenon scales. The English and German CIPS were factor analyzed resulting in a three factor theoretically preferred model aligned to Clance's (1985) original conceptualization of the impostor phenomenon as Fake, Luck and Discount (Holmes et al., 1993; Chrisman et al., 1995; Brauer and Wolf, 2016). However, a two factor model was shown to have a better statistical fit when compared to the three factor solution (Chrisman et al., 1995). Of interest, the English CIPS studies excluded four items from the 20-item CIPS to achieve a three factor model and the rationale was based on the results of a frequently referenced (but currently unobtainable) unpublished dissertation by Kertay et al. (1992). Despite the use of a 16-item CIPS which resulted in a theoretically preferred three factor model, the 20-item one factor model of the CIPS continues to be used and a total score is calculated for individuals who complete the measure. By comparison, the HIPS has been validated thrice using factor analysis for scale validation resulting in a two factor model (Edwards et al., 1987; Hellman and Caselman, 2004) and a third time, for construct validation against the fear of success/failure resulting in a four factor model (Fried-Buchalter, 1992). Similar to the CIPS, an overall total score continues to be calculated on the HIPS rather than subscale scores to reflect the multidimensional definition of the measure as postulated by Harvey (1981). Further to this, validation of the PFS has resulted in a two factor-solution of Inauthenticity and Self-deprecation as dimensions of Perceived Fraudulence, yet scoring of the measure involves an overall total score much like the CIPS and HIPS. Since the LIS is a unidimensional measure of the impostor phenomenon it was considered logical to calculate an overall impostorism score.

These results raise points for discussion about the conceptualization and dimensionality of impostorism. The CIPS, HIPS and PFS are based on multidimensional definitions of the impostor phenomenon, however, the measures are operationalized in research and applied settings by calculating overall scores, rather than subscale scores based on the corresponding factors. This evidence suggests a definitive conclusion about the dimensionality of the impostor phenomenon is unclear. Scoring of these measures appears to reflect a unidimensional conceptualization of the construct despite factor analysis results that indicate multiple dimensions. Clearer and convincing rationale would provide greater clarity about this methodology in scoring. Furthermore, additional evidence from future validation studies will aid in establishing item homogeneity or heterogeneity to ascertain the dimensionality of the impostor phenomenon.

#### Ascertaining a Gold Standard

Criterion validity was problematic in the selected studies because a clear ‘gold standard’ measure could not be ascertained. The quality appraisal tool required convincing arguments for a gold standard to be “gold” and a correlation coefficient with the gold standard equal to or greater than 0.70 (Terwee et al., 2007). This review has highlighted a gold standard is yet to be established and this is due to a number of factors relating to chronology, dimensional clarity and scale popularity.

In earlier studies, it was not possible to provide gold standard comparisons since the HIPS (Harvey, 1981) was the first impostor phenomenon measure developed and the CIPS was not constructed until 1985 for comparison to be possible. Five of the selected studies compared various impostor phenomenon measures to each other and reported consistently high correlation coefficients exceeding .70. A comparison of the CIPS and HIPS utilizing an ANCOVA, appeared to identify the CIPS as more sensitive in distinguishing between high and low impostorism in the non-clinical sample when CIPS scores were held constant (Holmes et al., 1993). It was also argued the CIPS had reduced incidents of false positives and false negatives when establishing cut-off scores. Researchers acknowledged that participants were not randomly assigned to groups, however, it was suggested the CIPS is the instrument of choice for use with the general population due to its sensitivity and reliability (Holmes et al., 1993). A comparison of the CIPS and PFS suggests the CIPS has better utility because of its brevity in comparison to the 51-item PFS. However, problematic interpretations of correlation coefficients did not provide convincing arguments to establish discriminant validity of the impostor phenomenon with other constructs and to assert the strengths of the CIPS over the PFS (Chrisman et al., 1995). Furthermore, instances of low internal consistency for the HIPS and the questionable stability of impostorism as measured by 14 items in a one factor model, rather than 11 items in a better fitting two factor solution did not provide convincing arguments for the HIPS being the gold standard either (Hellman and Caselman, 2004). These findings did not provide sufficient evidence to establish the CIPS nor HIPS as a gold standard measure of the impostor phenomenon. In addition, the PFS and LIS also require further validation to establish its strengths and utility as a potential gold standard measure. The limited number of PFS and LIS studies in this review demonstrated the need for further exploration of these measures.

An argument could be made the CIPS is the ‘gold standard’ measure by virtue of it being the most commonly cited and utilized measure by practitioners and impostor phenomenon researchers. However, popularity is not necessarily a reflection of higher quality. It would be premature to classify the CIPS as the gold standard measure of the impostor phenomenon in light of the results from this review. There remains to be questions about the dimensionality of the impostor phenomenon and its operationalization in measures such as the CIPS, HIPS, and PFS. Despite being based on multidimensional definitions of the construct, these measures calculate overall total scores and do not define subscale scores. Scoring of these measures appears to contradict the theoretical conceptualization of the impostor phenomenon. Without robust and consistent validation results and conceptual clarity, it is currently premature to select a ‘gold standard’ measure within the context of an evidence base that is still growing.

### Limitations

#### Search Strategy

The search strategy did result in the inclusion of two unpublished dissertations. Systematic reviews typically limit searches to peer-reviewed journal articles, however, due to the limited number of validation studies of impostor phenomenon measures a consensus decision was made by the researchers to include these dissertations. Following examination of reference lists and noticing frequent citations of these studies, it was justified to include the original scale development study for the HIPS and an often cited dissertation examining the construct validation of the HIPS (Harvey, 1981; Topping, 1983).

#### Quality Assessment Framework

The quality assessment framework used to appraise the measurement properties of the selected studies was originally designed for evaluating self-report questionnaires of officially diagnosable health conditions (Terwee et al., 2007). As expected, assessment criteria for specific methodological and psychometric properties are strict to distinguish between quality measures as they relate to patient reported health outcomes. However, the impostor phenomenon is not an official clinical condition, yet its measures are similar to self-report health measures and used to identify people and the degree to which they experience this phenomenon. Since impostorism is also linked to poorer well-being and poorer mental health outcomes (e.g., Chrisman et al., 1995; Sonnak and Towell), it was deemed appropriate to use this rigorous assessment framework and to tailor the application of specific criteria to studies to better reflect the current state of impostor phenomenon research. For example, the criteria to calculate Cronbach's alpha per dimension for internal consistency was only applicable to studies that conceptualized impostorism as a multidimensional construct. Despite these adjustments, specific psychometric properties did not necessarily receive higher scores and in some instances, it was not possible, however, it is emphasized these lower ratings are not necessarily a reflection of a poor study, but rather an absence of data or a current gap in the overall impostor phenomenon literature. The comprehensive reporting of essential data was a crucial element for studies to be appraised with higher scores based on the measurement criteria. A recommendation for improved reporting of data is made for future research. Also, the limited number of studies identified in this review, highlight the opportunity for increased research validating measures of impostorism and utilizing rigorous criteria to aid in research design and reporting, similar to established clinical conditions.

An alternative assessment framework was also considered for the present review—the Consensus-Based Standards for the Selection of Health Measurement Instruments (COSMIN) checklist—however, the criteria in this appraisal tool was considered too strict to assess the methodological quality of current impostor phenomenon studies (Mokkink et al., 2010; Terwee et al., 2012). The COSMIN checklist was also originally designed for the evaluation of Health-Related Patient-Reported Outcomes measures. Since impostorism is not an established diagnosable health condition and a definitive conclusion about its dimensionality has not been ascertained, applying an appraisal tool such as the COSMIN checklist may have resulted in much lower ratings that were not truly representative of the quality of current impostorism validation research.

### Recommendations for Future Directions

#### Longitudinal Stability

This review focused on trait measures of the impostor phenomenon. Therefore, measures based on definitions that conceptually assume consistencies in thoughts, feelings and behaviors across situations and time. Despite this theoretical assumption, research is yet to examine the longitudinal variability of impostorism measures, as was the case for all studies in this review. As a result, each study was allocated “not reported” ratings for reproducibility properties. Of interest, the HIPS, CIPS and PFS were examined in relation to “reflected” vs. “self-appraisals” (Leary et al., 2000). However, despite examining the test-retest reliability of the appraisal measures at the 6 to 8-week follow-up, the impostor phenomenon measures were not revisited to obtain longitudinal data. Rationale was not provided for this research design and highlights a consistent gap in the current evidence base. Similarly, another study examining undergraduates' impostorism feelings, self-esteem, personality, and predicted marks were collected at the beginning of the semester. However, at follow-up 1 week prior to an exam and after exam grades were released, longitudinal CIPS data was not collected alongside measures of self-esteem and questions about attributions of success or failure (Cozzarelli and Major, 1990). It is evident there is scope to improve research design in order to explore changes over time detected by trait impostor phenomenon scales. Currently, the developmental trajectory of the impostor phenomenon is unknown. Validation studies are yet to examine the longitudinal variability of impostorism scores. It is recommended future research explores the longitudinal stability and intensity of such scores. It is also suggested that researchers who examine change scores at a minimum, ensure means, standard deviations, and sample sizes are reported to enable sample size calculations.

#### Essential Psychometric Data

Based on the quality assessment framework, measurement gaps were identified in each study resulting in indeterminate or poor ratings across one or more criteria. Terwee et al. (2007) assert measures may be given many indeterminate or low ratings across measurement properties that are yet to be evaluated, especially if they are new questionnaires or due to less comprehensively reported validation studies. This subsequently results in lower ratings that are not necessarily due to poor questionnaire design or performance, but rather an absence of data or existing research. This was the case for several studies in this review, for example, the majority of studies demonstrated appropriate content validity and adequate internal consistency. However, due to doubtful design, no Item Response Theory or factor analysis, studies were then allocated indeterminate ratings for the internal consistency criterion. To better understand the dimensionality of the construct and therefore establish conceptual clarity of the impostor phenomenon, it is suggested statistical analyses utilizing Classical Test Theory and/or Item Response Theory are applied to impostorism measures with appropriate sample sizes. The comprehensive reporting of essential psychometric data will enable meaningful conclusions.

The floor and ceiling effects criterion, along with interpretability were also indeterminate, poor or largely unavailable for most studies since descriptive statistics were often omitted. The reporting of means and standard deviation scores from the measure of interest for at least four relevant subgroups is stipulated for interpretability, in order to assign qualitative meaning to quantitative data (Terwee et al., 2007). However, a limited number of studies provided this information. It was also common among the studies reviewed for sample descriptive statistics such as age not being reported or only age range (Harvey, 1981; Topping, 1983; Cozzarelli and Major, 1990; Fried-Buchalter, 1992; Leary et al., 2000). Gender distribution was also omitted in Harvey's (1981) original scale development study. It is assumed such data was collected, however, without the publication of this information, it is challenging for researchers and practitioners to make meaningful interpretations of findings and to select the most appropriate measure for specific purposes and populations. In the absence of essential data where practitioners require robust psychometrics to evaluate the utility of a measure, for example, for therapeutic settings, there is an increased risk of false positives and misidentifying those experiencing the impostor phenomenon.

Comprehensive reporting of psychometric data is necessary to assess the hypothesized structure of a construct and to evaluate the adequacy of its operationalization in measurement scales. Conducting this review also highlighted challenges in accessing research from the impostor phenomenon literature. While it was acknowledged comprehensive scale development data for some measures were only accessible upon request from first authors (Kolligian and Sternberg, 1991; Kertay et al., 1992; Leary et al., 2000), in most instances, attempts to obtain research data and/or papers in a timely manner were unsuccessful. This highlighted an important point about improving accessibility to scale development and validation information. Increasing the collection and publication of results will allow the scientific community to easily evaluate the quality and utility of impostor phenomenon instruments for future investigative and applied purposes, as will the trend in making research datasets publicly available.

#### Implications for Research and Applied Settings

This review has identified different conceptualizations of the impostor phenomenon and the measures associated with these definitions. There is a great deal of variability in the methodological quality and findings available to establish the dimensionality of the construct and therefore questions around what it is, what characteristics it is made up of and whether a gold standard instrument exists. For the purposes of research and applied settings, a clear purpose, target population and definition of the construct is necessary to select the most appropriate measure for its intended use. Availability of an established psychometrically sound gold standard measure of impostorism is also likely to be useful in related clinical areas of research where patient populations fear not meeting an inferred “audience” standard that they assume will result in negative evaluation; such populations also discount corrective information including the non-occurrence of feared outcomes. This is particularly the case for anxiety disorders featuring social threat fears such as social anxiety disorder (Rapee and Heimberg, 1997) and generalized anxiety disorder (American Psychiatric Association, 2013), though beliefs about being negatively evaluated by others also features in depression (Swann et al., 1992). A rigorous review of psychometric properties and justification for the use of a specific scale is always necessary. However, since the developmental trajectory of the impostor phenomenon is currently unknown, the use of such measures especially with child, adolescent, and older populations requires theoretically driven justification even more so. At least until greater understanding is established of the longitudinal variability of impostorism scores across the ages.

## Conclusions

Extensive variability in the methodological quality of impostorism validation studies currently exists. This review identified a gold standard measure is yet to be established and this has been limited by conceptual clarity around the dimensionality of the impostor phenomenon, its operationalization across measures, distributional properties across different groups (e.g., clinical samples, gender, age, cultures) and it's reproducibility. Quality ratings identified longitudinal research as an area for future directions and the need for consistent reporting of essential psychometric data to aid researcher and practitioner purposes. If scores are to be trusted as true representations of the impostor phenomenon, sufficient evidence of adequate validity, reliability and responsiveness of measures are necessary. This will enhance conceptual clarity and, the quality of scale development and validation studies.

## Author Contributions

KM conducted this systematic review as part of a Ph.D. thesis, and thus led the review. SK and MA provided supervision and ongoing advice regarding all aspects of the manuscript.

### Conflict of Interest Statement

The authors declare that the research was conducted in the absence of any commercial or financial relationships that could be construed as a potential conflict of interest.
